# Identification of two subtilisin‐like serine proteases engaged in the degradation of recombinant proteins in *Nicotiana benthamiana*

**DOI:** 10.1002/1873-3468.14014

**Published:** 2020-12-11

**Authors:** Alejandro A. Puchol Tarazona, Daniel Maresch, Annette Grill, Janet Bakalarz, Juan A. Torres Acosta, Alexandra Castilho, Herta Steinkellner, Lukas Mach

**Affiliations:** ^1^ Department of Applied Genetics and Cell Biology University of Natural Resources and Life Sciences Vienna Austria; ^2^ Department of Chemistry University of Natural Resources and Life Sciences Vienna Austria

**Keywords:** biopharmaceutical, molecular farming, monoclonal antibody, *Nicotiana benthamiana*, recombinant protein expression, serine protease

## Abstract

The tobacco variant *Nicotiana benthamiana* has recently emerged as a versatile host for the manufacturing of protein therapeutics, but the fidelity of many recombinant proteins generated in this system is compromised by inadvertent proteolysis. Previous studies have revealed that the anti‐HIV‐1 antibodies 2F5 and PG9 as well as the protease inhibitor α_1_‐antitrypsin (A1AT) are particularly susceptible to *N*. *benthamiana* proteases. Here, we identify two subtilisin‐like serine proteases (NbSBT1 and NbSBT2) whose combined action is sufficient to account for all major cleavage events observed upon expression of 2F5, PG9 and A1AT in *N. benthamiana*. We propose that downregulation of NbSBT1 and NbSBT2 activities could constitute a powerful means to optimize the performance of this promising platform for the production of biopharmaceuticals.

**Databases:**

NbSBT sequence data are available in the DDBJ/EMBL/GenBank databases under the accession numbers MN534996 to MN535005.

## Graphical abstract

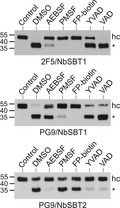

## Abbreviations

**A1AT**, α_1_‐antitrypsin

**Ac**, acetyl

**CDR**, complementarity‐determining region

**CHO**, aldehyde

**CMK**, chloromethylketone

**FMK**, fluoromethylketone

**FP**, fluorophosphonate

**mAb**, monoclonal antibody

**Nb**, *Nicotiana benthamiana*

**SBT**, subtilisin‐like serine protease

**Suc**, methoxysuccinyl

**Z**, benzyloxycarbonyl

The market for recombinant therapeutic proteins is increasing rapidly [1]. Plants have proved to be competent platforms for the production of biopharmaceuticals as demonstrated by the growing number of plant‐made therapeutic proteins being approved for human treatment or entering clinical trials [2, 3]. A plant particularly well suited for the rapid large‐scale synthesis of vaccines and monoclonal antibodies (mAbs) is the tobacco‐related species *Nicotiana benthamiana* [4] due to its high susceptibility to agrobacterium‐mediated transgene delivery by viral‐based expression vectors [5]. Furthermore, strategies have been developed which make it possible to express therapeutic proteins with human‐like glycans and other tailored post‐translational modifications in this plant [6, 7]. These technologies have been exploited for the manufacturing of the Ebola‐neutralizing antibody cocktail ZMapp [8] and the production of a seasonal influenza vaccine [9].

Despite all these qualities of *N. benthamiana* as an emerging plant‐based expression platform, a major drawback is still unresolved: recombinant proteins expressed in this plant frequently suffer from proteolytic degradation [10]. Several recombinant proteins with therapeutic potential including mAbs and human α_1_‐antitrypsin (A1AT) have been shown to undergo inadvertent proteolysis in *N. benthamiana* [11, 12, 13, 14]. This degradation reduces the yield as well as the quality of the protein of interest and can lead to erroneous conclusions about its size and other features [15]. Although proteases populate virtually all compartments of plant cells [10, 16], convincing evidence has been provided that the degradation of mAbs in *Nicotiana* species occurs primarily in the apoplast, the pericellular space in plant tissues [17]. Unfortunately, mAbs – like many other therapeutic proteins – need to be glycosylated for proper function and therefore have to be targeted to the secretory pathway [18].

Several strategies were proposed to counteract unwanted protein degradation in plants: targeted disruption of protease genes, coexpression of protease inhibitors and downregulation of endogenous protease activities by means of RNA interference or other antisense‐based mechanisms [19, 20]. For either strategy, substantial knowledge about the host enzymes involved in proteolytic breakdown of foreign proteins is required *a priori*. So far, the responsible proteases have not been identified in any plant‐based expression system, thus prohibiting targeted approaches to tackle unwanted proteolysis in *N. benthamiana* and other plant species.

Although the secretome of *N. benthamiana* is known to harbour proteases from all catalytic classes [16], we have previously shown that serine protease inactivators could block the antibody‐degrading capacity of *N. benthamiana* apoplastic fluid *in vitro* [11]. A recent study reported the presence of several subtilisin‐like serine proteases (SBTs) in the apoplast of agroinfiltrated *N. benthamiana* leaves [16]. SBTs constitute a large family of plant serine proteases with formidable endopeptidase activities [21]. In the present investigation, we aim to assess whether SBTs are involved in recombinant protein degradation in *N. benthamiana*. First, we isolate *N. benthamiana* SBTs present in the leaf apoplast using activity‐based probes and identify them by mass spectrometry. We then characterize the two most abundant enzymes (NbSBT1 and NbSBT2) and demonstrate their capacity to cleave mAbs and A1AT *in vitro*. In combination, NbSBT1 and NbSBT2 generate all cleavage products observed upon production of these potential biotherapeutics in plants. Hence, our results implicate NbSBT1 and NbSBT2 in recombinant protein degradation *in planta*.

## Materials and methods

### Isolation and identification of apoplastic subtilisin‐like serine proteases

*Nicotiana benthamiana* ∆XT/FT plants deficient in N‐glycan α1,3‐fucosylation and β1,2‐xylosylation [22] were grown for 4–5 weeks at 24 °C with a 16‐h light: 8‐h dark photoperiod. Concentrated apoplastic fluid was prepared as described [11] and then incubated for 1 h at 37 °C with 10 µm fluorophosphonate (FP)‐biotin (Santa Cruz Biotechnology, Santa Cruz, CA, USA). The sample (2.5 mL) was then chromatographed on a PD‐10 column (GE Healthcare, Chicago, IL, USA) equilibrated in 100 mm sodium acetate (pH 5.5). The recovered eluate (3.5 mL) was incubated with 40 µL avidin‐agarose beads (Sigma‐Aldrich, St. Louis, MO, USA) for 16 h at 4 °C under constant agitation. The beads were washed four times with 4 mL 100 mm sodium acetate (pH 5.5) and once with 4 mL 10 mm Tris/HCl (pH 6.8) prior to elution of the bound proteins with 80 µL SDS/PAGE sample buffer (5 min, 95 °C). The samples were then subjected to 12.5% SDS/PAGE under reducing conditions prior to staining of the gel with Coomassie Brilliant Blue (CBB) R‐250. The 63–70 kDa bands were excised, S‐carboxamidomethylated and then digested with sequencing‐grade trypsin (Promega, Madison, WI, USA). The peptides thus generated were fractionated on a Thermo BioBasic C18 separation column (5 µm particle size, 150 × 0.32 mm) operated using a Dionex UltiMate 3000 system (Thermo Fisher Scientific, Waltham, MA, USA). A gradient from 96.5% solvent A and 3.5% solvent B (A: 65 mm ammonium formate, pH 3.0; B: 80% acetonitrile, 20% A) to 40% B in 45 min was applied, followed by a 15‐min gradient from 40% B to 95% B, at a flow rate of 6 µL·min^−1^. Eluted peptides were analysed online on a maXis 4G ETD Q‐TOF mass spectrometer (Bruker, Billerica, MA, USA) equipped with an electrospray ionization source and operated in the positive ion mode (*m/z* range: 150‐2200).

### Molecular cloning of subtilisin‐like serine protease cDNAs

Total RNA was extracted from 35‐mg samples of various *N. benthamiana* tissues using the SV Total RNA Isolation Kit (Promega). First‐strand cDNA was synthesized from 1 µg RNA using the RevertAid H Minus First Strand cDNA Synthesis Kit (Thermo Fisher Scientific) and oligo(dT)_18_ as primer. PCR fragments encompassing the complete ORF of the identified apoplastic SBTs were obtained from total leaf or flower cDNA using suitable primer combinations (Table S1) and then subcloned into pCR4‐TOPO (Thermo Fisher Scientific) or pBluescript II KS (+) (Agilent Technologies, Santa Clara, CA, USA). The fidelity of the cloned cDNAs was verified by automated DNA sequencing (Microsynth, Balgach, Switzerland).

For ectopic expression of the two most abundant apoplastic SBTs (NbSBT1 and NbSBT2) in *N. benthamiana*, the coding sequences of these enzymes were PCR‐amplified using Q5 High‐Fidelity DNA polymerase (New England Biolabs, Ipswich, MA, USA) and then transferred by Gibson assembly [23] into pEAQ‐IgA‐hc [24] after digestion of the vector with AgeI and XhoI to remove its previous insert. After sequence confirmation, both constructs were transformed into *Agrobacterium tumefaciens* strain UIA143 [12].

### Production and purification of recombinant NbSBT1 and NbSBT2

Leaves of 5‐week‐old *N. benthamiana* plants were inoculated with agrobacterial suspensions diluted to an OD_600_ of 0.15 with infiltration buffer (10 mm MES, 10 mm MgSO_4_, 0.1 mm acetosyringone, pH 5.6). Three days after infiltration, the leaves were submerged in extraction buffer (50 mm sodium phosphate/200 mm KCl, pH 7.0) prior to vacuum exposure in a desiccator. Exogenous buffer was removed prior to centrifugation of the leaves for 15 min at 2000 ***g*** and 4 °C. The recovered exudate was then supplemented with 20 mm imidazole and loaded on a 1 mL column of Chelating Sepharose (GE Healthcare) charged with Ni^2+^ ions. After washing with the same buffer, bound proteins were eluted with 250 mm imidazole in extraction buffer. Protein‐containing eluate fractions were pooled, dialysed twice against 2 L of extraction buffer and then concentrated by ultrafiltration using Microsep Advance centrifugal devices (molecular weight cut‐off: 10 kDa; Pall Corporation, New York, NY, USA).

### *In vitro* degradation assays

mAbs (50–200 µg·mL^−1^; Polymun Scientific, Klosterneuburg, Austria) or human plasma A1AT (50 µg·mL^−1^; Sigma‐Aldrich) were incubated with concentrated apoplastic fluid (10–250 µg·mL^−1^), NbSBT1 (1–30 µg·mL^−1^) or NbSBT2 (3–70 µg·mL^−1^) in 100 mm sodium acetate (pH 5.5) at 37 °C in the absence or presence of selected protease inhibitors (Sigma‐Aldrich or Bachem, Bubendorf, Switzerland). After incubation for 1–16 h, samples were mixed with an equivalent volume of SDS/PAGE sample buffer and heated at 95 °C for 5 min prior to analysis by western blotting with antibodies to human IgG (Sigma‐Aldrich) or A1AT (Abcam, Cambridge, UK) as described previously [11, 12]. Streptavidin–peroxidase (Vector Laboratories, Burlingame, CA, USA) was used for the detection of proteins labelled with FP‐biotin or biotinyl‐YVAD‐CMK (Bachem) on western blots [25]. To isolate mAb degradation products, reaction mixtures (500 µL) were mixed with 10 µL rProtein A Sepharose 4 Fast Flow beads (GE Healthcare) and incubated for 2 h at 4 °C under constant shaking. The beads were collected by centrifugation and washed four times with PBS. Elution of bound proteins from the beads was achieved using 0.1 m glycine/HCl (pH 3.0). Protein‐containing eluate fractions were immediately neutralized by addition of 0.1 m Tris/HCl (pH 8.0), subjected to buffer‐exchange into phosphate‐buffered saline and then concentrated by ultrafiltration using Amicon YM30 centrifugal filter units (Merck Millipore, Burlington, MA, USA).

### Cleavage site analysis

N‐terminal sequence analysis of bands blotted on polyvinylidene difluoride membranes (Bio‐Rad Laboratories, Hercules, CA, USA) was performed by Edman degradation on an Applied Biosystems Procise 492 protein sequencer (Protein Micro‐Analysis Facility, Medical University of Innsbruck, Austria) as described previously [11]. Alternatively, antibodies or their degradation products were treated with PNGase F (Roche, Basel, Switzerland) to release their N‐glycans, reduced with 5 mm DTT (45 min, 56 °C) and then fractionated on a Thermo ProSwift RP‐4H column (250 × 0.20 mm) using a Dionex UltiMate 3000 HPLC system (Thermo Fisher Scientific). Native and digested A1AT samples were analysed without prior deglycosylation and reduction. After application of the sample (5 µL), elution was performed at 65 °C and a flow rate of 8 µL·min^−1^ with a gradient of 20–95% solvent B (80% acetonitrile in 0.01% trifluoroacetic acid) in solvent A (0.05% trifluoroacetic acid) over 40 min as follows: 20–65% B (15 min), 65–95% B (5 min). Eluted polypeptides were analysed online on a maXis 4G ETD Q‐TOF mass spectrometer (Bruker) equipped with an electrospray ionization source and operated in the positive ion mode (*m/z* range: 400–3800). The analysis files were deconvoluted (Maximum Entropy Method) using DataAnalysis 4.0 (Bruker) and manually annotated.

## Results

### Identification of subtilisin‐like serine proteases present in the *N. benthamiana* leaf apoplast

**Fig. 1:**
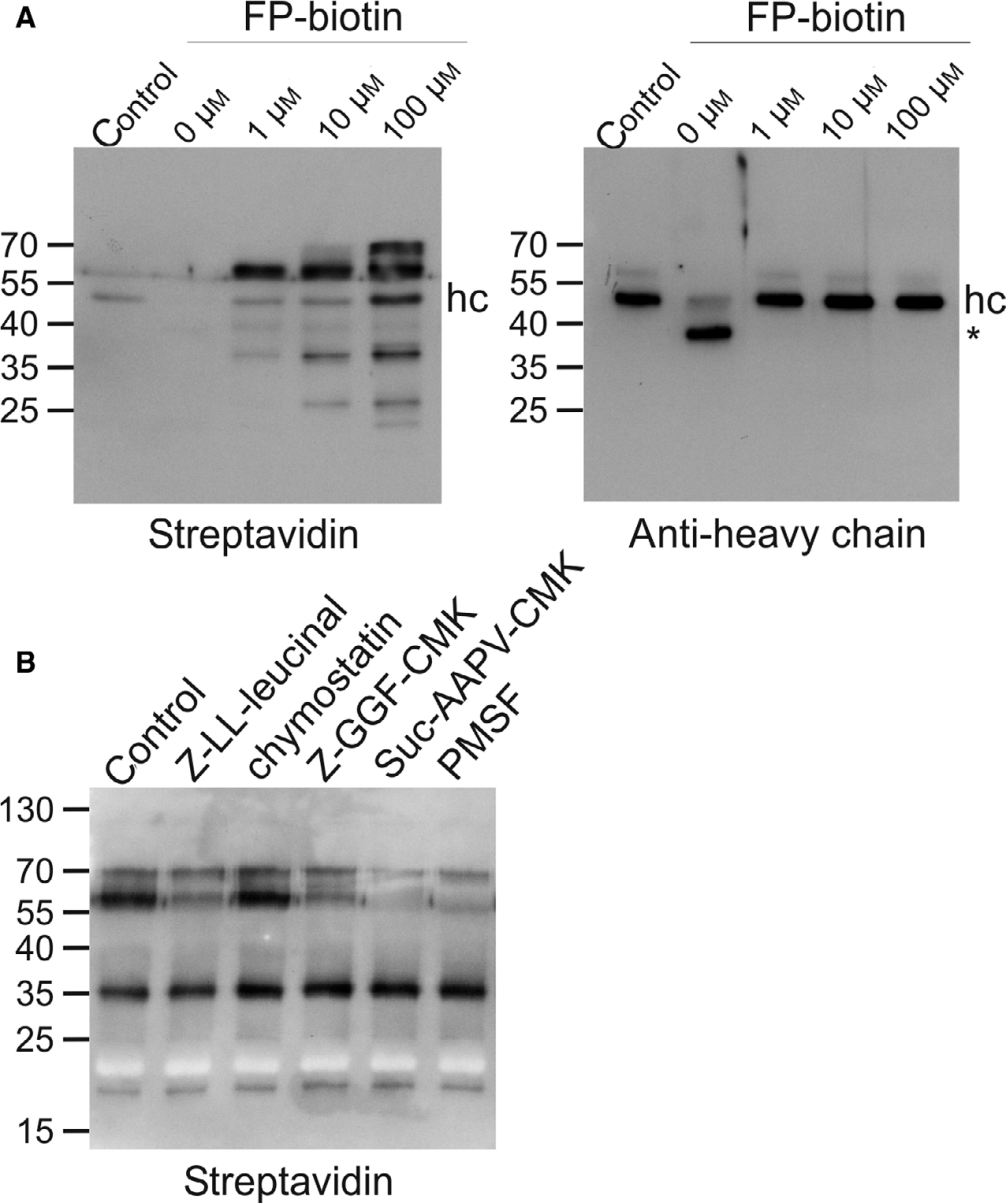
(A) Reactivity of apoplastic proteins with FP‐biotin. Apoplastic fluid (85 µg total protein/mL) was pretreated with the indicated concentrations of FP‐biotin (1 h, 37 °C) prior to incubation with 200 µg·mL^−1^ mAb 2F5 (4 h, 37 °C). Untreated 2F5 was loaded as a control. Samples were analysed by SDS/PAGE and western blotting using streptavidin–peroxidase or peroxidase‐labelled antibodies to the heavy chain of human IgG for detection. Note the nonspecific binding of FP‐biotin and streptavidin–peroxidase to the heavy chain of 2F5. hc, full‐length heavy chain; *, 40 kDa heavy chain degradation product. (B) Effect of serine protease inhibitors on the labelling of apoplastic proteins with FP‐biotin. Apoplastic fluid was pretreated with DMSO (control) or the indicated protease inhibitors (100 μm Z‐LL‐leucinal; 100 μm chymostatin; 100 μm Z‐GGF‐CMK; 100 µm Suc‐AAPV‐CMK; 1 mm PMSF) for 30 min at 37 °C prior to labelling with 10 µm FP‐biotin (4 h, 37 °C). Samples were analysed by SDS/PAGE followed by western blotting with streptavidin–peroxidase as detection reagent. The migration positions of selected molecular mass standards are indicated, with their respective masses expressed in kDa.

To identify the repertoire of SBTs present in the *N. benthamiana* leaf apoplast, apoplastic fluid collected from untreated *N. benthamiana* leaves was incubated with FP‐biotin, a biotinylated activity‐based probe which potently reacts with serine hydrolases [26]. Its FP moiety attaches covalently to active‐site serine residues, allowing the probe to discriminate between catalytically competent and inactive forms of the targeted enzymes. Treatment of apoplastic fluid with FP‐biotin abolished processing of the proteolysis‐sensitive anti‐HIV‐1 mAb 2F5 even at low probe concentrations, indicating that FP‐biotin efficiently inactivates the protease(s) in action. Western blot analysis with streptavidin–peroxidase revealed that FP‐biotin‐labelled apoplastic polypeptides accumulated in a number of discrete bands in the size range of 15–70 kDa. The most intensely labelled band corresponded to polypeptide(s) of 63–65 kDa, which showed pronounced reaction with FP‐biotin even at a probe concentration as low as 1 µm (Fig. 1A). To identify bands containing serine proteases, apoplastic fluid was incubated with various synthetic serine protease inhibitors prior to addition of FP‐biotin. This pretreatment interfered most strongly with the labelling of the 63–65 kDa band, with methoxysuccinyl (Suc)‐Ala‐Ala‐Pro‐Val‐chloromethylketone (Suc‐AAPV‐CMK) and phenylmethylsulphonyl fluoride (PMSF) being the most effective compounds. Notably, preincubation with Suc‐AAPV‐CMK also reduced the labelling of the 67–70 kDa band (Fig. 1B).

FP‐labelled proteins were isolated by affinity chromatography using immobilized avidin and then fractionated by preparative SDS/PAGE. The 63–65 kDa and 67–70 kDa bands were excised, subjected to tryptic digestion and analysed by mass spectrometry to determine the identities of the constituent proteins. In good agreement with a similar activity‐based profiling analysis of the secretome of agroinfiltrated *N. benthamiana* leaves [16], 10 different SBTs could be unanimously identified by the detection of at least two unique peptides (Table S2). The coding sequences of these SBTs were cloned from reverse‐transcribed *N. benthamiana* RNA and found to closely match those predicted from the most current *N. benthamiana* gene assemblies [27, 28].

### Ectopic expression and purification of recombinant *N. benthamiana* SBTs

**Fig. 2:**
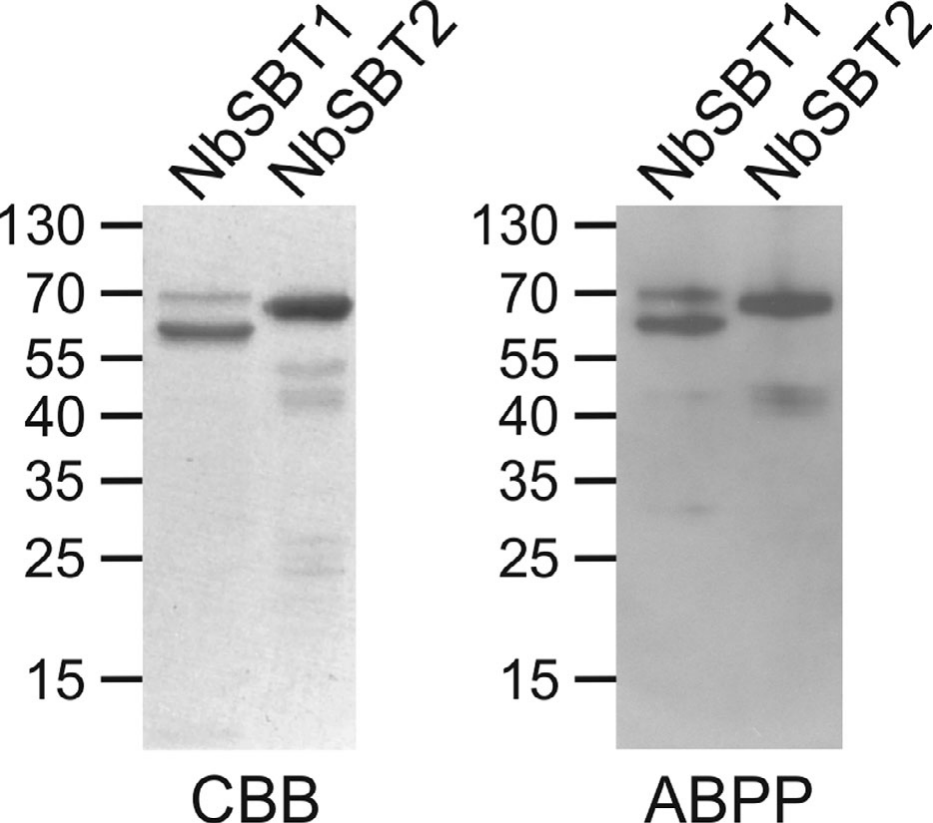
Characterization of purified NbSBT1 and NbSBT2. Hexahistidine‐tagged NbSBT1 and NbSBT2 produced in *N. benthamiana* were purified by metal‐chelate affinity chromatography and then analysed by SDS/PAGE under reducing conditions followed by staining with CBB. For ABPP, purified NbSBTs were incubated with 100 µm FP‐biotin (NbSBT1) or 10 μm biotinyl‐YVAD‐CMK (NbSBT2) for 1 h at 37 °C prior to analysis by SDS/PAGE under reducing conditions and western blotting using streptavidin–peroxidase for detection. The migration positions of selected molecular mass standards are indicated, with their respective masses expressed in kDa.

As judged from spectral counts and sequence coverage by mass spectrometry (Tables S3,S4 and Figs S1,S2), we selected the two most abundant SBTs present in the affinity‐purified samples (NbSBT1 and NbSBT2) for further studies. Hexahistidine‐tagged versions of NbSBT1 and NbSBT2 were expressed in *N. benthamiana* leaves and then purified by nickel‐chelate affinity chromatography. Purification of an empty‐vector control (EV) (Fig. S3) was performed using leaves inoculated with the parental expression vector. SDS/PAGE under reducing conditions of purified NbSBT1 and NbSBT2 revealed a complex band pattern consistent with autocatalytic processing (Fig. 2), a phenomenon reported previously for other plant SBTs [29]. Bands of 67–70 kDa were observed in both samples, which agrees well with the predicted molecular mass of mature NbSBT1 and NbSBT2 after removal of the prodomain (69.5 kDa without accounting for potential N‐glycosylation). Both 67–70 kDa bands were subjected to tryptic fingerprinting by mass spectrometry. The most N‐terminal peptide detected was in either case a semitryptic fragment. The sequences of these two semitryptic peptides (NbSBT1: T^110^THSWDFLK, NbSBT2: T^111^THTSQFLGLNSK) correspond to the predicted N termini of the respective mature peptidase domains. As determined by Edman degradation, the major 63 kDa band observed in the NbSBT1 sample was found to be an N‐terminally truncated form of mature NbSBT1 starting with G^207^ (Table S2).

To assess its activity status, purified NbSBT1 was treated with FP‐biotin. Since NbSBT2 is closely related to *Nicotiana tabacum* phytaspase which selectively cleaves after aspartic acid residues [30], the activity‐based probe biotinyl‐Tyr‐Val‐Ala‐Asp‐chloromethylketone (biotinyl‐YVAD‐CMK) was utilized in this instance. As expected, the 67–70 kDa forms of NbSBT1 and NbSBT2 were found to be catalytically competent. Interestingly, the 63 kDa form of NbSBT1 was also reactive with FP‐biotin. Since one residue of the catalytic triad (Asp^148^) is located within the fragment removed during proteolytic processing, these results indicate that NbSBT1 is cleaved into a two‐chain form consisting of an N‐terminal light chain (T^110^‐A^206^) and a C‐terminal heavy chain (G^207^‐N^770^) without losing its enzymatic activity (Fig. S4). Hence, the heavy chain of NbSBT1 could account for the 63–65 kDa band observed upon labelling of apoplastic proteins with FP‐biotin (Fig. 2).

### Cleavage of recombinant proteins by NbSBT1 and NbSBT2 *in vitro*

**Fig. 3:**
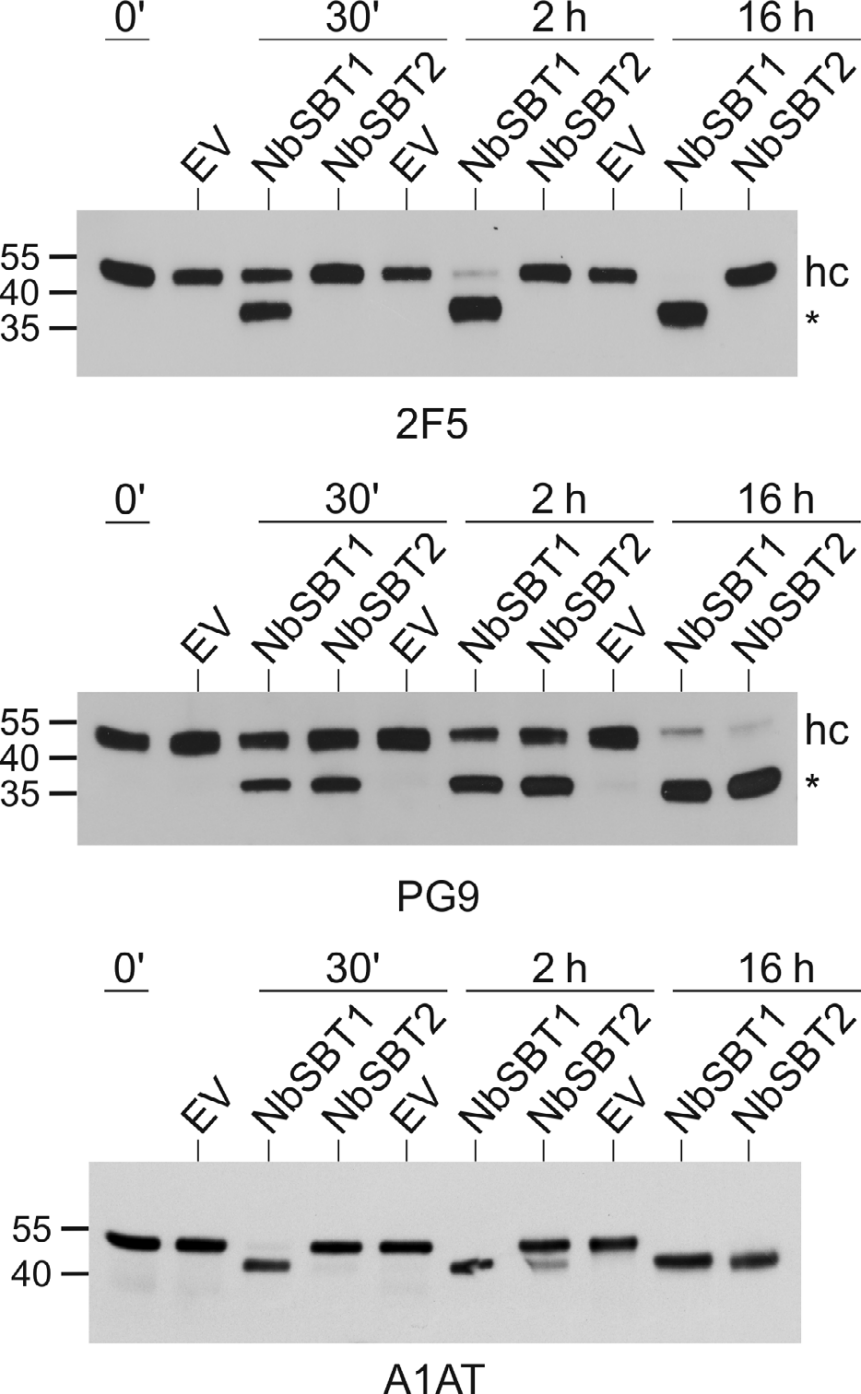
Processing of mAbs and A1AT by NbSBT1 and NbSBT2 *in vitro*. mAbs (2F5, PG9) or A1AT (50 µg·mL^−1^) were incubated with purified NbSBT1 (2 µg·mL^−1^), NbSBT2 (4 µg·mL^−1^) or an equivalent volume of EV at pH 5.5 for the indicated times and then analysed by immunoblotting with antibodies to the heavy chain of human IgG (2F5, PG9) or A1AT, respectively. The migration positions of selected molecular mass standards are indicated, with their respective masses expressed in kDa. hc, full‐length heavy chain; *, 40 kDa heavy chain degradation product.

To assess the potential of NbSBT1 and NbSBT2 for protein degradation *in planta*, we first tested their capacity to cleave the mAbs 2F5 and PG9 – two broadly neutralizing human IgG1 antibodies to HIV‐1 that undergo substantial proteolytic degradation upon their production in the leaves of *N. benthamiana* [11]. Each mAb was incubated with NbSBT1 or NbSBT2 at pH 5.5 to simulate apoplastic conditions. Upon exposure to NbSBT1, the heavy chains of 2F5 and PG9 were cleaved yielding characteristic 40 kDa bands. These fragments resemble the heavy chain degradation products produced *in planta* and found to originate from cleavage within, or next to, the complementarity‐determining region (CDR) H3 loop [11]. Interestingly, NbSBT2 did not process 2F5. Nevertheless, the latter enzyme was capable of cleaving the heavy chain of PG9, generating also a 40 kDa product as observed for NbSBT1 (Fig. 3).

In order to identify the cleavage sites, the 2F5 and PG9 degradation fragments were purified by protein A affinity chromatography and then subjected to both N‐terminal sequencing and analysis by mass spectrometry. NbSBT1 cleaved 2F5 at only one position: besides the intact light chain, only the two heavy chain fragments arising from cleavage at TTLF^107^↓G^108^VP were detected by mass spectrometry. In agreement with this result, Edman degradation of the 40 kDa 2F5 fragment revealed a single N terminus starting with G^108^. 2F5 is cleaved at the same position when the antibody is produced *in planta* or upon incubation of the antibody with leaf apoplastic fluid *in vitro* [11]. In the case of PG9, NbSBT1 processed the heavy chain at YNYY^111^↓D^112^FY, YNYH^121^↓Y^122^MD, YHYM^123^↓D^124^VW, TTVT^133^↓V^134^SS and VTVS^135^↓S^136^AS. Cleavage at Y^111^↓D^112^ is documented for PG9 extracted from plants [11]. Incubation of PG9 with NbSBT2 resulted in proteolysis of the heavy chain at NYYD^112^↓F^113^YD, DFYD^115^↓G^116^YY and HYMD^124^↓V^125^WG, demonstrating a notable preference of NbSBT2 for aspartic acid residues at the substrate position preceding the scissile bond (Table S5; Fig. S5). Hydrolysis after D^112^, D^115^ and D^124^ was also observed in PG9 isolated from plants or upon incubation of the antibody with apoplastic fluid [11, 31].

A1AT is another example of a recombinant protein prone to proteolytic inactivation when expressed in *N. benthamiana* [12]. When A1AT was incubated with NbSBT1, rapid proteolysis into a 40 kDa form was observed. Two new C termini were identified (E^378^ and M^382^), both located in the reactive centre loop of the protease inhibitor. Truncation of A1AT *in planta* occurs largely by cleavage at the same positions [12]. NbSBT2 acted on A1AT far slower than NbSBT1, possibly owing to the absence of aspartic acid residues in the reactive centre loop (Fig. 3; Table S5).

**Fig. 4:**
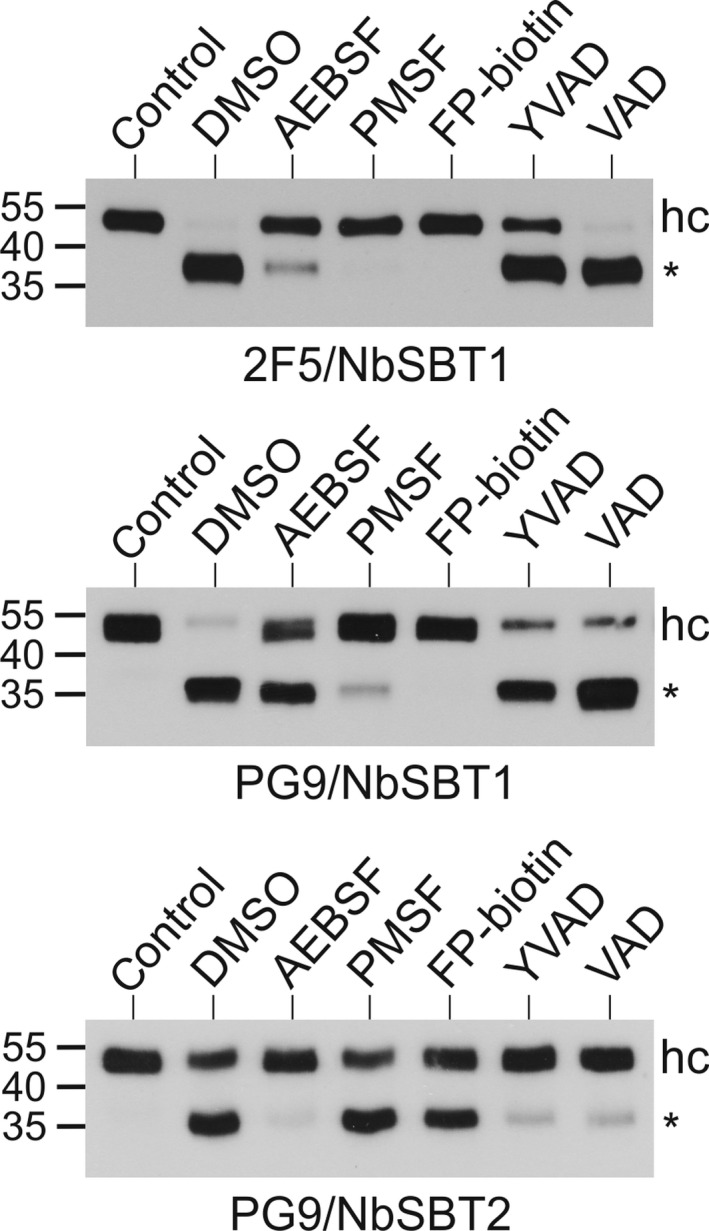
Effect of protease inhibitors on *in vitro* processing of mAbs. 2F5 or PG9 (50 µg·mL^−1^) were incubated with purified NbSBT1 (2 µg·mL^−1^) or NbSBT2 (4 µg·mL^−1^) for 3 h after preincubation of the enzymes with solvent (DMSO) or the indicated protease inhibitors [20 mm AEBSF; 1 mm PMSF; 100 µm FP‐biotin; 100 µm Ac‐YVAD‐CMK (YVAD); 100 µm Z‐VAD‐FMK (VAD)] for 1 h at 37 °C. Samples were then analysed by immunoblotting with peroxidase‐labelled antibodies to the heavy chain of human IgG. Untreated mAbs were loaded as controls. The migration positions of selected molecular mass standards are indicated, with their respective masses expressed in kDa. hc, full‐length heavy chain; *, 40 kDa heavy chain degradation product.

While *in vitro* cleavage of 2F5 by apoplastic fluid can be completely prevented by the addition of PMSF or FP‐biotin, processing of PG9 is most effectively inhibited by a combination of aminoethylbenzenesulphonyl fluoride (AEBSF) and acetyl (Ac)‐YVAD‐CMK [31]. We have therefore tested the effects of these inhibitors on the degradation of 2F5 and PG9 by NbSBT1 and NbSBT2 (Fig. 4). FP‐biotin and PMSF proved highly reactive with NbSBT1: they were the most effective NbSBT1 inactivators, almost completely blocking proteolysis of both mAbs. AEBSF also showed a substantial inhibitory capacity. By contrast, Ac‐YVAD‐CMK and benzyloxycarbonyl‐Val‐Ala‐Asp‐fluoromethylketone (Z‐VAD‐FMK) were much less effective. The inhibition profile of NbSBT2 differed. Ac‐YVAD‐CMK and Z‐VAD‐FMK were the substances most effective at inactivating NbSBT2, almost completely preventing the cleavage of PG9. This high sensitivity to Ac‐YVAD‐CMK and Z‐VAD‐FMK is in agreement with the substrate specificities of NbSBT2 homologues from tobacco and tomato [29, 30]. AEBSF also showed a strong inhibitory capacity towards NbSBT2. By contrast, PMSF and FP‐biotin were far less effective. These data support the notion that NbSBT1 accounts for most 2F5 proteolysis in *N. benthamiana*, whereas both NbSBT1 and NbSBT2 are involved in the degradation of PG9. In line with previously published inhibition profiles [12], NbSBT1 seems to be largely responsible for A1AT processing, although NbSBT2 could play a minor role in this process.

## Discussion

Although many studies have reported instances of unintended proteolysis in plant‐based expression systems [10, 19], efforts to identify proteases involved in these degradation processes have proved futile so far. We have now isolated two *N. benthamiana* SBTs which are capable of cleaving two proteolysis‐prone mAbs (2F5, PG9) as well as another protease‐susceptible human serum glycoprotein (A1AT) at the same positions as it happens *in planta* or upon incubation with unfractionated apoplastic fluid. Together, the activities of NbSBT1 and NbSBT2 are sufficient to account for all inadvertent proteolytic events which occur during production of these potential biotherapeutics in *N. benthamiana* [11, 12]. These results suggest that NbSBT1 and NbSBT2 are the key enzymes involved in processing of mAbs and A1AT *in planta*. However, we cannot rule out that other apoplastic SBTs contribute to mAb and A1AT cleavage in *N. benthamiana*. The documented catalytic redundancy of plant SBTs [21, 32] suggests that some of the other apoplastic SBTs identified in this study could display similar substrate specificities as NbSBT1 and NbSBT2.

Proteomic studies have provided comprehensive evidence that infiltration with agrobacteria modifies the proteolytic landscape of *N. benthamiana* [16]. Hence, it has been proposed that the process of agroinfiltration triggers the synthesis of the proteases involved in heterologous protein degradation in this plant. However, NbSBT1 and NbSBT2 are abundant apoplastic proteins present in similar amounts in agroinfiltrated and untreated *N. benthamiana* leaves. Moreover, activity‐based probe profiling (ABPP) confirmed that these two proteases are catalytically active in the apoplast under both conditions [16]. These findings are fully consistent with our observation that agroinfiltration does not affect the capacity of apoplastic fluid to process the mAbs 2F5 and PG9 *in vitro* [11]. However, it is possible that other *N. benthamiana* SBTs are upregulated upon infection with agrobacteria and thus enhance the proteolytic potential of apoplastic fluid after agroinfiltration. It should be noted that agroinfiltration has been shown to upregulate the expression of papain‐like cysteine proteinases and legumains [33], which have been also implicated in the degradation of recombinant proteins in *N. benthamiana* and related *Nicotiana* species [11, 20, 34].

Although NbSBT1 is capable of processing the tomato immune protease Rcr3 [35], the physiological functions of NbSBT1 and NbSBT2 are currently unknown. Still, some assumptions can be made based on studies of their homologues in other plant species including *Arabidopsis thaliana* and *N. tabacum*. The *A. thaliana* SBT most similar to NbSBT1 (recently also named NbSBT5.2 [35]) is AtSBT5.2, with whom it shares 58% sequence identity. As noted for NbSBT1, AtSBT5.2 is capable of autocatalytic processing [36]. Originally, AtSBT5.2 was identified as CO_2_ response secreted protease (CRSP). This CO_2_‐induced proteolytic activity mediates the repression of stomata formation upon exposure of *A. thaliana* leaves to elevated atmospheric CO_2_ concentrations. Mechanistically, AtSBT5.2 has been shown to process epidermal patterning factor 2 (EPF2), the precursor of an extracellular peptide repressing stomatal development [37]. However, the proposed cleavage site of AtSBT5.2 in EPF2 (SLPD↓CSYA) displays no close resemblance with the sequences hydrolysed by NbSBT1 in 2F5, PG9 and A1AT. AtSBT5.2 is also capable of processing the precursor of inflorescence deficient in abscission (IDA), yielding the mature IDA peptide involved in the abscission of floral organs [32]. This cleavage event occurs at the sequence YLPK↓GVPI. Interestingly, the four residues following the scissile bond are identical to those of the NbSBT1 cleavage site in the CDR H3 loop of 2F5 (TTLF↓GVPI).

NbSBT2 is a close relative of *N. tabacum* phytaspase, a constitutively secreted apoplastic SBT promoting programmed cell death in response to viral infection or exposure to abiotic stresses [38]. Like NbSBT2, phytaspase has an exquisite preference for aspartic acid residues at P1, the position preceding the scissile bond [30]. Similar observations were made for *Solanum lycopersicum* Phyt2 [29], which displays 86% sequence identity with NbSBT2 and thus probably represents its tomato orthologue. Phyt2 is capable of cleaving the precursor of the wound hormone systemin at two positions (VRED↓LVAQ and MQTD↓NNKL), which results in the release of a biologically active processing intermediate [39]. Generation of the abscission‐inducing peptide phytosulphokine by Phyt2 is due to precursor maturation at AHLD↓YIYT [40]. These results compare well with NbSBT2, which also tolerates many amino acids at substrate positions other than P1. Phytaspase is potently inhibited by peptide aldehydes such as Ac‐YVAD‐CHO and Ac‐VAD‐CHO [38]. This is in good agreement with the efficient inactivation of NbSBT2 by Ac‐YVAD‐CMK and Z‐VAD‐FMK. By contrast, these two inhibitors exert comparatively small effects on the proteolytic activity of NbSBT1. On the other hand, PMSF is a far better inactivator of NbSBT1 than of NbSBT2. Hence, the combined application of PMSF and Ac‐YVAD‐CMK could present a powerful means to prevent unwanted proteolysis of recombinant proteins in extracts from *N. benthamiana* as long as lines with silenced or disrupted NbSBT1 and NbSBT2 genes are not available.

## Author contributions

LM conceived and supervised the study; AP, DM, AG, JB, JT, AC and LM designed experiments; AP, DM, AG, JB, JT and LM performed experiments; AP, DM and LM analysed data; AC and HS provided key reagents; AP and LM wrote the original draft of the manuscript; AP, DM, HS and LM made manuscript revisions.

## Supporting information


**Table S1.** Oligonucleotide primers used in this study.
**Table S2.** Sequences of apoplastic NbSBTs.
**Table S3.** NbSBT1 peptides identified by mass spectrometry.
**Table S4.** NbSBT2 peptides identified by mass spectrometry.
**Table S5.** Cleavage sites of NbSBT1 and NbSBT2 in 2F5, PG9 and human α_1_‐antitrypsin (A1AT).
**Fig. S1.** Location of the identified peptides in the NbSBT1 sequence.
**Fig. S2.** Location of the identified peptides in the NbSBT2 sequence.
**Fig. S3.** Absence of subtilisin‐like serine proteases in empty‐vector controls.
**Fig. S4.** Proteolytic processing of NbSBT1 and NbSBT2.
**Fig. S5.** Processing of 2F5 and PG9 by NbSBT1 and NbSBT2.
