# Supplementary material for: Identification of two subtilisin‐like serine proteases engaged in the degradation of recombinant proteins in Nicotiana benthamiana
Source: FEBS Lett. 2020 Dec 11;595(3):379–88. doi: 10.1002/1873-3468.14014 (PMC8221030; doi:10.1002/1873-3468.14014)
Supplement: Supplementary file 1 — Table S1. Oligonucleotide primers used in this study. Table S2. Sequences of apoplastic NbSBTs. Table S3. NbSBT1 peptides identified by mass spectrometry. Table S4. NbSBT2 peptides identified by mass spectrometry. Table S5. Cleavage sites of NbSBT1 and NbSBT2 in 2F5, PG9 and human α1‐antitrypsin (A1AT). Fig. S1. Location of the identified peptides in the NbSBT1 sequence. Fig. S2. Location of the identified peptides in the NbSBT2 sequence. Fig. S3. Absence of subtilisin‐like serine proteases in empty‐vector controls. Fig. S4. Proteolytic processing of NbSBT1 and NbSBT2. Fig. S5. Processing of 2F5 and PG9 by NbSBT1 and NbSBT2. [file FEB2-595-379-s001.pdf]

Supporting Information

Identification of two subtilisin-like serine proteases engaged in the degradation of recombinant proteins in *Nicotiana benthamiana*

Alejandro A. Puchol Tarazona[1], Daniel Maresch[2], Annette Grill[1], Janet Bakalarz[1], Juan A. Torres Acosta[1], Alexandra Castilho[1], Herta Steinkellner[1], Lukas Mach[1]

[1]Department of Applied Genetics and Cell Biology, University of Natural Resources and Life Sciences, Muthgasse 18, A-1190 Vienna, Austria
[2]Department of Chemistry, University of Natural Resources and Life Sciences, Muthgasse 11, A-1190 Vienna, Austria

Correspondence: Dr. Lukas Mach, Department of Applied Genetics and Cell Biology, University of Natural Resources and Life Sciences, Muthgasse 18, A-1190 Vienna, Austria.
E-mail: lukas.mach@boku.ac.at

Table S1. Oligonucleotide primers used in this study.

NbSBT1_NotI_fw	5’-ctaggcggccgcttcatcaatatttgtcacgg-3’
NbSBT1_EcoRI_rev	5’-tgagaattctctgaaaatgttccccttg-3’
NbSBT2_fw	5’-tcccatacctctcactcctc-3’
NbSBT2_rev	5’-caagagcttagtgcaccgtc-3’
NbSBT3_fw	5’-gcttatgttatagaaattatccc-3’
NbSBT3_rev	5’-tcataatcagttctgtcctc-3’
NbSBT4_PstI_fw	5’-gatactgcagcatttcttgctcatccattc-3’
NbSBT4_NotI_rev	5’-gcagatgcggccgcgctataggctatgtcatacc-3’
NbSBT5_fw	5’-ttacctgacactgagaac-3’
NbSBT5_rev	5’-gtacatttgcagtgaatgac-3’
NbSBT6_NotI_fw	5’-ctaggcggccgcgttcttgtattaatttgcagc-3’
NbSBT6_KpnI_rev	5’-aatcggtacctcatggaatcacaaacaatc-3’
NbSBT7_EcoRI_fw	5’-gatgaattccttctctttcaaaccctacac-3’
NbSBT7_KpnI_rev	5’-aatggtacctcgatcaaaacaacaggatc-3’
NbSBT8_EcoRI_fw	5’-gcagagaattcgccaaacacacagcaacc-3’
NbSBT8_NotI_rev	5’-ttatagcggccgctacatttgactccgagc-3’
NbSBT9_EcoRI_fw	5’-cgagcgaattcatcttcactcccatcactc-3’
NbSBT9_NotI_rev	5’-gcagatgcggccgcattcgaaactcgagatatcg-3’
NbSBT10_fw	5’-tttggaggtgtctgaagag-3’
NbSBT10_rev	5’-ttggataaagcaaatgccg-3’
NbSBT1_Gibson_fw	5’-gttaagcttctgtatattctgcccaaattcgcgatgaaaggaattatatctttg-3’
NbSBT1_Gibson_rev	5’-gaaaatttaatgaaaccagagttaaaggcctagtgatggtgatggtgatggttgctacttacgacg-3’
NbSBT2_Gibson_fw	5’-gttaagcttctgtatattctgcccaaattcgcgatggcaaatcatattaccttg-3’
NbSBT2_Gibson_rev	5’-gaaaatttaatgaaaccagagttaaaggcctagtgatggtgatggtgatggttgtgtcctgtcaaag-3’

Table S2. Sequences of apoplastic NbSBTs. Unique tryptic peptides identified by mass spectrometry are highlighted in red. Active-site residues are underlined and displayed with a yellow background. The observed processing sites in recombinant NbSBT1 and NbSBT2 are indicated by red arrows.

NbSBT1:
MKGIISLFFCFSLFIVSFILREADSASQAQNNGIYIVYMGAAASSNGGTRHDQARLISSLIRRNKHAVVHS
YNNGFSGFAARLSESEAKSMAQRPGVISVFPDPVLQLH↓TTHSWDFLKYQTDEKINSSPSSGSDSSLIGA
DTIIGILDTGIWPESESFNDKDMGPIPSRWNGTCMDGQDFGSSKCNKKIVGARFYEESDDSGTKIA↓GSA
RDENGHGTHVASTAAGSPVAGASYYGLAAGTATGGSPGSRISMYRVCTTFGCRGSAIMKAFDDAIADG
VDVLSLSLGSSPGLEPDFPSNPIAIGAFHAVEKGITVVCSAGNSGPGPKTVVNTAPWILTVAATTIDRDFETDI
VLGGNKLIKGGGINFGNMTKSSVYPLIHGNSTKSNDNVSEADARSCVPGSLDENKVKGKIVLCENLDD
GEYFPSDKLDEVKSRGGVGFILIDDDERTVAPKFNSFSAGVVSKKDGNEILAYINSTRNPVASILPTVSITKYKPAP
VVAYFSSRGPAYNTPNLLKPDITAPGVAILAAWPGNDTSEALPGQKPPIFNLLSGTSMSCPHVSGIAATVKA
VNPTWSPSAVKSAIMTTAIQTNNLKAPITTVSGSKATPYDIGAGEASTSGPLKPGLVYETDVADYLQFLCSV
GFNISQIKLISITVPEDFSCPKNSTSELVSNMNYPSIAISSLKENEPKKVTRTVTNTGEEASVYTTVIEAPKG
LEVQVIPTKLEFTNKSKKLSYDVSFKASSTSKEDLFGSITWTNGKYKVRSPFVVSSN

NbSBT2:
MANHITLCIWLLFFFISIFSLAKSETYIIHMDLSAMPKAFSSHHNWYLTTLSSVSDSSTNHKDFLSSKLVY
AYTNAIHGFSASLSPSELEAIKYSPGYVSSIKDISVKID↓TTHTSQFLGLNSKSGVWPTSDYGKDIIIGLVDTGI
WPESKSYSDDGISEVPSRWKGECESGTEFNSSLCNKKLIGARYFNKGLLANNPNLNISMNSSRDTDGHGTHT
SSTAAGSYVEGASYFGYATGTAIGIAPKAHVAMYKALWEEGVYLSDVLAAIDQAITDGVDVLSLSLGID
AIPLHEDPVAIAAFAALEKGIFVSTSAGNEGPYYETLHNGTPWVLTVAAGTVDREFIGTLTLGNGVSVPG
LSLYPGNSSSSESSLVYVECQDDKELQKNAHKFVVCLDKNDSVGEHVYNVRNSKVAGAVFITNTTDLE
FYLQSEFPAVFLNLQEGDKVLEYIKSNSAPKGKLEFQVTHIGAKPAPKVATYSSRGPSPSCPSILKPDLM
APGALILASWPQQSPVTDVTSGKLFSNFNIISGTSMSCPHASGVAALLKAAHPEWSPAAIRSAMMTTSTALD
NTQSPIRDIGNKNAAATPLAMGAGHIDPNKALDPGLIYDATPQDYVNLLCALNFTSKQIKTITRSSSYTC
SNPSLDLNYPSFIGFFNGNSSESDPKRIQEFKRTVTNLQDGTSIYTANFTPMGKFKVSVVPEKLVFKEKYEKLS
YKLRIEGPIVMDDNVVYGSLSWVETGGNYVVRSPIVATSIKVDPLTGHN

NbSBT3:
MANCITMYFLLLTILLLTLNPLTMAESETYIIHMDLSAMPKAFSSHQNWYLTTLASVSGSSSLGTESNRN
SFSSSKLVYAYTNAIHGFSATLSPSELQVIKNSPGYLSSTKDMTVKIDTTHTSQFLGLNSDSGAWPKSDYGKDV
IVGLVDTGIWPESKSYNDNGMTEVPSRWKGECESGTQFNSSLCNKKLIGARYFNKGLIANNPNITISMNSAR
DTDGHGTHTSSTAAGSHVESASYFGYARGSATGMAPKAHVAMYKALWEEGTMLSDILAAIDQAIEDG
VDIISLSLGIDDLALYEDPVAIATFAAMEKDIFVSTSAGNEGPDDQALHNGTPWVLTVAAGTVDREFIGT
LSLGNGVSVTGLSLYPGNSSSSESSIVFLKTCLEEKELEKNAHKFAVCYDTNGSVSDQLYNVKNTKVAG
GIFITNYTDLEFYLQSEFPAVFLNFEDGDKVLEYIKNSHSPKARLEFQVTHLGAKPAPKVASYSSRGPSES
CPFILKPDLMAPGALILASWPQKSPATQIRSGELFSNFNIISGTSMSCPHAAGVAALLKGAHPKWSPAAIR
SAMMTTADTMDNMQMPIRDIGRNNNAASPLAMGAGRINPNKALDPGLIYDITSQDYINLLCALDFTSQ
QIKAITRSSAYSCSNSSLDLNYPSFIGYFNYNSSESDPKRIQEFQRTVTNVGEGMSVYTAKLTSMGDYKASVA
PDKLVFKEKYEKQSYKLRIEGPLLVDNYLVYGSLSWVETSGKYVVKSPIVATTIRVDPL

NbSBT4:
MSRYPVILVVVALLFLCRLSVAMMKKKTYIIHMAKSQMPATFNDDHTHWYDSSLRSVSDSAEMLYVY
NNVVHGFSARLTPQEVESLETQPGILSVLPDLKYQLHTTRTPTFLGIDKSADFFPESDAMSDVIIGVLDTG
VWPESKSFDDSGLGPIPASWKGQCESGTNFSSSNCNRKLIGARYFSRGYETTLGPIDESKESKSPRDDDG
HGTHTSTTAGGSVVQGANLFGYAPGTARGMATRARVAVYKVCWVGGCFSSDILAAMDKAIDDNVNV
LSLSLGGGISDYYRDSVAIGAFAAMEKGILVSCSAGNAGPSPYSLSNVAPWITTVGAGTLDRDFPAYVSL
GNGKNFSGVSLYKGNSLPNKMLPFVYAGNASNVTNGNLCMTGTLIPEKVKGKIVLCDRGINARVQKGS
VVKAAGGAGMVLTNTAANGEELVADAHLLPATAVGQKTGDAIRDYLTSDSNPTATILFEGTKVGIEPSPV
VAAFSSRGPNSITPEILKPDIIAPGVNILAGWTGAVGPTGLEEDDRRVGFNIISGTSMSCPHVSGLAALLKGA
HPEWSPAAIRSALMTTAYTSYKNGGAIQDVSTGKRSTPFDHGAGHVDPVSALNPGLVYDITADDYLNFLCA
LDYTPSQISSLARRNFTCNESKKYSVTDLNYPSFAVSFPAESSARTGSAGSSSIKYSRTLTNVGPAGTYKV
TVTSPSTSSVKITVEPETLSFSQMNEKKSYTVTFTAPSMSSSTTNVFGRIEWSDGKHVVGSPVAISWT

NbSBT5:
MARPGGMVLSTLFLMLFHVFVHAGQNQKKTYIIYMDKSNIPADFDDHTLWYDSSLKSVSKGANMLYT
YNNVIHGYSTQLTADEAKSLEQQPGILSVHEEVRYELHTTRSPTFLGLEGRESKSFFLQAETRSEVIIGVL

DTGVWPESKSFDDTGLGPVPMSWKGECQIGKNFKASSCNRKLIGARFFSQGYEAAFGAIDETTESKSPR
DDDGHGTHTATTAAGSVVTGASLFGYAAGTARGMASHARVAAYKVCWAGGCFSSDILAGMDQAVID
GVNVLSLSLGGTISDYYRDIVAIGGFSAASQGIFVSCSAGNGGPGSGSLSNAAPWITTVGAGTMDREFPA
YISLGNGKKFSGVSLYSGKALPSSVMPLVYAGNASQASNGNLCTSGSLIPEKVDGKIVVCDRGMNARA
QKGLVVKDAGGIGMILANTDSYGDELVADAHLIPTAAVGQTAGDLIKRYIASDSNPTTTIAFGGTKLGVQ
PSPVVAAFSSRGPNPITPEILKPDLIAPGVNILAGWTGKVGPTGLPEDTRNVGFNIISGTSMSCPHVSGLAALL
KAAHPEWSPAAIRSALMTTGYSTYKNGKMIEDVATGMSSTPFDHGAGHVNPAAALNPGLVYDLTVDD
YINFLCALDYSPSMIKVIAKRDISCENNKEYRVADLNYPSFAIPLETAWGEHANSSAPTVTRYTRTLTNV
GNPATYKASVSSEMQEVKIQVEPQTLTFSRKKEKKTYTVTFTASSKPSGTTSFARLEWSDGQHVVASPI
AFSWT

NbSBT6:
MGFLQILLFCIVCSFPCPSFQTDLEIYIVQVEPPETQISTQSSSMDLESYYHSFLPKTTTISSSENEEPRMIYS
YHNVMKGFAARLTAAQVKEMEKKHGFVSAEPQRILSLHTTHTPSFLGLQQNMGLWKDSNYGKGVIIG
VLDTGIVPDHPSFSDVGMPPPPAKWKGFCESNFTTKCNNKLIGARSFPLDNGPIDENGHGTHTASTAAG
AFVKGANVFGNANGTAVGVAPLAHIAIYKVCGSDGVCSDVEILAAMDVAIDDGVDILSISLGGTSNPFH
NDKIALGAYSATERGILVSCSAGNSGPFQRTVDNDAPWILTVGASTHDRKLKATVKLGNKEEFEGESAY
HPKTSNSTFFTLFDVEKIVHEQPVAPFCIPGSLTDPSIRGKIVVCLVGGGVRTVDKGQVVKDAGGVGMIL
INNPEDGVTKSAEAHVLPALDVSDADGKKILAYINSTSNPVAAITFHGTVLGDKNAPIVASFSSRGPSEA
SRGILKPDIIGPGVNVLAAWPTSVDNNKNTKSTFNIISGTSMSCPHLSGIAALLKSVHPDWSPAAIKSAIM
TTTDTLNLAKNPILDERLIPADIFAIGAGHVNPSRANDPGLVYDTPFEDYVPYLCGLNYTNREVGKLLQG
KVNCSEVKSIPEAQLNYPSFSIRLRSTPQTYTRTVTNVGNATSIYKVEIVSPKGVAVKVKPSMLNFSVLN
QKLTYQVTFSKTTNNSNREVGQGFLKWNSNTHSVSSPIAVVLLVE

NbSBT7:
MASLLLLTHFLCFTFTAIIPIFSPVSAEPAAKTYIFRVDSFSKPAIFPTHYHWYSSEFTEAVNILHVYDNVF
HGFSASLSPSQAASILQHPSILAAFEDRRRQRHTTRSPQFLGLRNQKGLWSESDYGSDVIVGVLDTGIWP
ERRSFSDLNLGPVPTRWKGVCQTGDKFTAKNCNRKIIGARYFSKGHEAAPGFGGIGGGGINDTVEFKSP
RDADGHGTHTASTAAGRHAFSANMSGYASGIAKGVAPKARLAVYKVCWKNSGCFDSDILAAFDFAVS
DGVDVISISIGGGDGISSPYYLDPIAIGAYGAVSRGVFVSSSAGNDGPNGMSVTNLAPWLATVGAGTIDR
NFPAEVILGDGRKLSGVSLYAGKPLRGKMYPIVYPGKSGVLSASLCMENSLDPHLVRGKIVICDRGSNP
RVAKGMVVNKAGGVGMILANGVSNGEGLVGDAHLIPTCAVGANEGDAIKSYIASHPTASATINFHGTV
IGVKPAPVVASFSGRGPNGLNPEILKPDLIAPGVNILAAWTDAVGPTGLDLDNRKAEFNILSGTSMACPH
VSGAAALLKSAHPDWSPAAIRSAMMTTANRVDNRLQPMTDEATGKPATPYDYGAGHLNLDLALDPGL
VYDLANEDYVSFLCAIEYDPKTIQVITKSPVDCPMKKPLPENLNYPSIAALFSTAAKGVSSKTFFRTVTN
VGDTNVEYRVKIEAPKGVTVNVKPDKLVFSEKVRKLSYYVTITVDSKNLVLNDSGAVFGSLSWIDGKH
VVRSPIVVTQMSPL

NbSBT8:
MSRFTMLVVLVLVLLCLCHLSVATIGSSNKKSTYIVHVSKSQMPESFEDHKRWYDSSLKSVSDSAEML
YVYNNVVHGFSARLTVQEAESLERQSGILTVLPEMKYELHTTRTPSFLGLDRSADFFPESNAMSDVIVG
VLDTGVWPESKSFDDTGLGPVPDSWKGECESGTNFSSSNCNRKLIGARYFSKGYETILGPVDVSKESKS
ARDDDGHGTHTATTAAGSIVQRASLFGYASGTARGMATRARVAVYKVCWIGGCFSSDILAAMDKAID
DNVNVLSLSLGGGNSDYYRDSVAIGAFAAMEKGILVSCSAGNAGPSPYSLSNVAPWITTVGAGTLDRD
FPAYVSLGNGKNFSGVSLYKGDLSLSKMLPFVYAGNASNTTNGNLCMTGTLIPEKVKGKIVLCDRGINP
RVQKGSVVKEAGGVGMVLANTAANGDELVADAHLLPATTVGQTTGEAIKKYLTSDPNPTATILFEGTK
VGIKPSPVVAAFSSRGPNSITQEILKPDIIAPGVNILAGWTGAVGPTGLAEDTRRVGFNIISGTSMSCPHVS
GLAALLKGAHPEWSPAAIRSALMTTAYTVYKNGGALQDVSTGKPSTPFDHGAGHVDPVAALNPGLVY
DLRADDYLNFLCALNYTSIQINSIARRNYNCETSKKYSVTDLNYPSFAVVFPEQMTAGSGSSSSSVKYTR
TLTNVGPAGTYKVSTIFSPSNSVKVSVEPETLVFTRANEQKPYTVTFTAPSTPSTTNVFGRIEWSDGKHV
VGSPVAISWT

NbSBT9:
MSRFTMLVVLVLVLLCLCHLSVAITGSSSNKKSTYIVHVSNSQMPESFEDHTHWYDSSLKSVSDSAEML
YVYNNVVHGFSARLTVQEAESLERQSGILSVLPELKYELHTTRTPSFLGLDRSADFFPESNAMSDVIVGV
LDTGVWPESKSFNDTGLGPVPDSWKGECESGTNFSSSNCNRKLIGARYFSKGYETTLGPVDVSKESKSA
RDDDGHGTHTATTAAGSIVQGASLFGYASGTARGMATRARVAVYKVCWIGGCFSSDILAAMDKAIDD
NVNVLSLSLGGGNSDYYRDSVAIGAFAAMEKGILVSCSAGNAGPSPYSLSNVAPWITTVGAGTLDREFP
AYVSLGNGKNFSGVSLYKGDLSLSKMLPFVYAGNASNTTNGNLCMTGTLIPEKVKGKIVLCDRGINPR
VQKGSVVKEAGGVGMILANTAANGDELVADAHLLPATTVGQMTGEAIKKYLASDPNPTATILFEGTK
VGIKPSPVVAAFSSRGPNSITQEILKPDIIAPGVNILSGWTGAVGPTGLAEDTRRVRFNIISGTSMSCPHVS
GLAALLKGAHPDWSPAAIRSALMTTAYTVYKNGGALQDVSTRKPSTPFDHGAGHVDPVAALNPGLVY

DLRADDYLNFLCALNYTSIQINSIARRNYNCETSKKYSVTDLNYPSFAVVFPEQMTAGSGSSASSVKYT
RTLTNVGPAGTYKVSTVFSPSNSVKVLVEPESLVFTHANEQKSYTVTFTAPSTPSTTNVFGRIEWSDGKH
VVGSPVAISWT

NbSBT10:
MSGMCFGLVIVLLSGILHVGKAEIYIVTVEGEPVISYKGGIDGFEATASESDEKIDTTSELVTSYARHLEK
KHDMLLALLFDRGTYKKIYSYHHLINGFAAHISHEQAEILKQAPGVKSVERDWKVRRLTTHTPQFLGLP
TGVWPTGGGFDRAGEDIVIGFVDSGIYPHHPSFSSHNTEPYGPLPKYRGKCEVDPNTKKDYCNGKIIGA
QHFAEAAKAAGAFNPTVDYDSPIDGDGHGSHTAAIAAGNNGIPVRMHGFEFGRASGMAPRARIAVYK
ALYRLFGGFVADVVAAIEQAVRDGVDILNLSVGPNSPPATTKTTFLNPFDATLLSAVKAGVFVAQAAG
NGGPFPKTLLSYSPWIVSVAAAVDDRRYKNHLTLGNGKILPGIGLSPSTHPNRTFTMVAANDVLLDSSV
TKYSPADCQRPEVLNKNLVEGNILLCGFSFNFVVGTASIKKVAETAKALGAAGFVLAVENASPGAKFDP
VPVSIPGILITDVSQSMELVDYYNITTSRDWTGRVKSFKSTGSIGNGLRPILHKSAPQVAIFSARGPNIKDY
SFQDADLLKPDILAPGSLIWAAWAPNGTDEANFCGEGFALISGTSMAAPHIAGISALIKQHHPHWSPAAI
KSALMTTSSTIDRAERPLQAQQYSGSETMMLVPATPFDYGSGHVNPRAALDPGLIFDAGYQDYLGFLCT
VPGIDPHEIKNFTHTPCNYTLGHPSNFNSPSIAVAHLVGTRTITRTVINVAEEETYVITARMAPEIAIETIPP
AMTLRHGASRKFTVTLTVRAVTGAYSFGEVLLKGSRGHKVRIPVVAAGYDR

Table S3. NbSBT1 peptides identified by mass spectrometry. The total MASCOT score of the protein was 1096.5 (sequence coverage: 28.1%).

m/z meas.	Mass calc.	z	Score	Sequence	Range
500.9192	1499.7402	3	46.5	K.GITVVCSAGNSGPGPK.T	311 - 326
750.8486	1499.7402	2	50.3	K.GITVVCSAGNSGPGPK.T	311 - 326
750.8654	1499.7402	2	73.7	K.GITVVCSAGNSGPGPK.T	311 - 326
750.8714	1499.7402	2	93.1	K.GITVVCSAGNSGPGPK.T	311 - 326
750.8752	1499.7402	2	83.6	K.GITVVCSAGNSGPGPK.T	311 - 326
750.8772	1499.7402	2	87.8	K.GITVVCSAGNSGPGPK.T	311 - 326
681.4178	2041.1208	3	24.0	K.TVVNTAPWILTVAATTIDR.D	327 - 345
681.4179	2041.1208	3	27.5	K.TVVNTAPWILTVAATTIDR.D	327 - 345
681.4207	2041.1208	3	36.9	K.TVVNTAPWILTVAATTIDR.D	327 - 345
866.7119	2597.2207	3	49.6	K.IVLCENLDDGEYFPSDKLDEVK.S	411 - 432
703.2999	1404.6885	2	21.3	R.GGVGFILIDDDER.T	435 - 447
703.3351	1404.6885	2	50.4	R.GGVGFILIDDDER.T	435 - 447
703.3525	1404.6885	2	40.4	R.GGVGFILIDDDER.T	435 - 447
703.3532	1404.6885	2	51.9	R.GGVGFILIDDDER.T	435 - 447
703.3659	1404.6885	2	55.1	R.GGVGFILIDDDER.T	435 - 447
703.3668	1404.6885	2	52.9	R.GGVGFILIDDDER.T	435 - 447
703.3671	1404.6885	2	41.7	R.GGVGFILIDDDER.T	435 - 447
703.3676	1404.6885	2	54.5	R.GGVGFILIDDDER.T	435 - 447
703.3676	1404.6885	2	44.4	R.GGVGFILIDDDER.T	435 - 447
703.3680	1404.6885	2	59.9	R.GGVGFILIDDDER.T	435 - 447
703.3681	1404.6885	2	47.5	R.GGVGFILIDDDER.T	435 - 447
703.3736	1404.6885	2	40.5	R.GGVGFILIDDDER.T	435 - 447
703.3741	1404.6885	2	21.0	R.GGVGFILIDDDER.T	435 - 447
703.3743	1404.6885	2	31.5	R.GGVGFILIDDDER.T	435 - 447
703.3750	1404.6885	2	22.0	R.GGVGFILIDDDER.T	435 - 447
703.3755	1404.6885	2	58.1	R.GGVGFILIDDDER.T	435 - 447
703.3828	1404.6885	2	29.3	R.GGVGFILIDDDER.T	435 - 447
703.4113	1404.6885	2	38.9	R.GGVGFILIDDDER.T	435 - 447
703.8131	1404.6885	2	26.4	R.GGVGFILIDDDER.T	435 - 447
515.3584	514.3115	1	26.2	R.TVAPK.F	448 - 452
515.3700	514.3115	1	26.8	R.TVAPK.F	448 - 452
571.8170	1141.5768	2	74.9	K.FNSFSAGVVSK.K	453 - 463
571.8223	1141.5768	2	69.8	K.FNSFSAGVVSK.K	453 - 463
571.8243	1141.5768	2	69.5	K.FNSFSAGVVSK.K	453 - 463
571.8317	1141.5768	2	87.4	K.FNSFSAGVVSK.K	453 - 463
720.3989	1438.8395	2	23.6	R.NPVASILPTVSITK.Y	478 - 491
720.4350	1438.8395	2	64.1	R.NPVASILPTVSITK.Y	478 - 491
720.4405	1438.8395	2	57.0	R.NPVASILPTVSITK.Y	478 - 491
720.4418	1438.8395	2	62.8	R.NPVASILPTVSITK.Y	478 - 491
495.6064	1483.7823	3	31.9	K.YKPAPVVAYFSSR.G	492 - 504
495.6115	1483.7823	3	31.0	K.YKPAPVVAYFSSR.G	492 - 504
495.6144	1483.7823	3	41.3	K.YKPAPVVAYFSSR.G	492 - 504
495.9329	1483.7823	3	21.9	K.YKPAPVVAYFSSR.G	492 - 504
742.8666	1483.7823	2	61.3	K.YKPAPVVAYFSSR.G	492 - 504
742.8972	1483.7823	2	43.2	K.YKPAPVVAYFSSR.G	492 - 504
742.9136	1483.7823	2	45.4	K.YKPAPVVAYFSSR.G	492 - 504
742.9137	1483.7823	2	35.2	K.YKPAPVVAYFSSR.G	492 - 504
628.8407	1255.6561	2	49.3	K.AVNPTWSPSAVK.S	568 - 579
628.8429	1255.6561	2	38.8	K.AVNPTWSPSAVK.S	568 - 579
628.8444	1255.6561	2	42.7	K.AVNPTWSPSAVK.S	568 - 579
628.8515	1255.6561	2	40.6	K.AVNPTWSPSAVK.S	568 - 579

m/z meas.	Mass calc.	z	Score	Sequence	Range
753.3775	1504.7919	2	61.6	K.SAIMTTAIQTNNLK.A	580 - 593
753.3849	1504.7919	2	52.5	K.SAIMTTAIQTNNLK.A	580 - 593
753.3855	1504.7919	2	84.2	K.SAIMTTAIQTNNLK.A	580 - 593
753.3889	1504.7919	2	31.8	K.SAIMTTAIQTNNLK.A	580 - 593
753.3925	1504.7919	2	77.1	K.SAIMTTAIQTNNLK.A	580 - 593
753.3954	1504.7919	2	78.2	K.SAIMTTAIQTNNLK.A	580 - 593
753.3976	1504.7919	2	48.2	K.SAIMTTAIQTNNLK.A	580 - 593
753.4072	1504.7919	2	53.5	K.SAIMTTAIQTNNLK.A	580 - 593
753.9072	1504.7919	2	41.8	K.SAIMTTAIQTNNLK.A	580 - 593
761.3644	1520.7868	2	69.0	K.SAIMTTAIQTNNLK.A	580 - 593
761.8610	1520.7868	2	45.3	K.SAIMTTAIQTNNLK.A	580 - 593
480.7744	959.5288	2	45.6	K.APITTVSGSK.A	594 - 603
480.7809	959.5288	2	38.1	K.APITTVSGSK.A	594 - 603
480.7878	959.5288	2	47.7	K.APITTVSGSK.A	594 - 603
480.7889	959.5288	2	45.6	K.APITTVSGSK.A	594 - 603
960.6374	959.5288	1	31.8	K.APITTVSGSK.A	594 - 603
803.4016	1604.8120	2	23.1	K.LISITVPEDFSCPK.N	649 - 662
803.4067	1604.8120	2	39.4	K.LISITVPEDFSCPK.N	649 - 662
803.4105	1604.8120	2	23.6	K.LISITVPEDFSCPK.N	649 - 662
704.0010	2109.0477	3	32.1	R.TVTNTGEEASVYTTVIEAPK.G	693 - 712
704.0087	2109.0477	3	38.7	R.TVTNTGEEASVYTTVIEAPK.G	693 - 712
704.0109	2109.0477	3	42.8	R.TVTNTGEEASVYTTVIEAPK.G	693 - 712
704.0116	2109.0477	3	46.8	R.TVTNTGEEASVYTTVIEAPK.G	693 - 712
704.0139	2109.0477	3	31.8	R.TVTNTGEEASVYTTVIEAPK.G	693 - 712
704.0385	2109.0477	3	41.9	R.TVTNTGEEASVYTTVIEAPK.G	693 - 712
1055.4978	2109.0477	2	42.5	R.TVTNTGEEASVYTTVIEAPK.G	693 - 712
1055.4978	2109.0477	2	54.8	R.TVTNTGEEASVYTTVIEAPK.G	693 - 712
1055.5042	2109.0477	2	42.0	R.TVTNTGEEASVYTTVIEAPK.G	693 - 712
1055.5094	2109.0477	2	69.9	R.TVTNTGEEASVYTTVIEAPK.G	693 - 712
1055.5128	2109.0477	2	69.8	R.TVTNTGEEASVYTTVIEAPK.G	693 - 712
1055.5138	2109.0477	2	59.7	R.TVTNTGEEASVYTTVIEAPK.G	693 - 712
1055.9688	2109.0477	2	65.8	R.TVTNTGEEASVYTTVIEAPK.G	693 - 712
542.3413	1082.6336	2	54.8	K.GLEVQVIPTK.L	713 - 722
542.3423	1082.6336	2	41.2	K.GLEVQVIPTK.L	713 - 722
542.3531	1082.6336	2	59.8	K.GLEVQVIPTK.L	713 - 722
542.3567	1082.6336	2	56.0	K.GLEVQVIPTK.L	713 - 722
1083.6104	1082.6336	1	27.7	K.GLEVQVIPTK.L	713 - 722
908.4393	1815.0142	2	21.7	K.GLEVQVIPTKLEFTNK.S	713 - 728
908.4686	1815.0142	2	32.4	K.GLEVQVIPTKLEFTNK.S	713 - 728
908.4860	1815.0142	2	39.4	K.GLEVQVIPTKLEFTNK.S	713 - 728
751.4048	750.3912	1	25.0	K.LEFTNK.S	723 - 728
543.8083	1085.5757	2	49.0	K.KLSYDVSFK.A	731 - 739
543.8084	1085.5757	2	35.1	K.KLSYDVSFK.A	731 - 739
1086.5515	1085.5757	1	20.5	K.KLSYDVSFK.A	731 - 739
958.4399	957.4807	1	32.7	K.LSYDVSFK.A	732 - 739
958.4472	957.4807	1	26.7	K.LSYDVSFK.A	732 - 739
958.4717	957.4807	1	30.8	K.LSYDVSFK.A	732 - 739
958.4881	957.4807	1	22.4	K.LSYDVSFK.A	732 - 739
836.3911	835.4076	1	34.0	R.SPFVVSSN.-	763 - 770
836.4037	835.4076	1	21.4	R.SPFVVSSN.-	763 - 770
836.4106	835.4076	1	29.3	R.SPFVVSSN.-	763 - 770

Table S4. NbSBT2 peptides identified by mass spectrometry. The total MASCOT score of the protein was 998.7 (sequence coverage: 21.9%).

m/z meas.	Mass calc.	z	Score	Sequence	Range
598.7816	1195.5510	2	33.1	K.SGVWPTSDYGK.D	124 - 134
598.7859	1195.5510	2	43.4	K.SGVWPTSDYGK.D	124 - 134
598.7984	1195.5510	2	42.7	K.SGVWPTSDYGK.D	124 - 134
598.8055	1195.5510	2	42.9	K.SGVWPTSDYGK.D	124 - 134
878.4482	1754.9455	2	58.2	K.DIIIGLVDTGIWPESK.S	135 - 150
878.4515	1754.9455	2	55.5	K.DIIIGLVDTGIWPESK.S	135 - 150
878.4537	1754.9455	2	57.8	K.DIIIGLVDTGIWPESK.S	135 - 150
878.4557	1754.9455	2	46.5	K.DIIIGLVDTGIWPESK.S	135 - 150
706.2712	1410.6263	2	48.8	K.SYSDDGISEVPSR.W	151 - 163
706.2814	1410.6263	2	59.4	K.SYSDDGISEVPSR.W	151 - 163
706.3038	1410.6263	2	59.1	K.SYSDDGISEVPSR.W	151 - 163
706.3096	1410.6263	2	59.1	K.SYSDDGISEVPSR.W	151 - 163
706.3106	1410.6263	2	53.5	K.SYSDDGISEVPSR.W	151 - 163
706.3110	1410.6263	2	51.1	K.SYSDDGISEVPSR.W	151 - 163
706.3153	1410.6263	2	49.8	K.SYSDDGISEVPSR.W	151 - 163
706.3228	1410.6263	2	44.1	K.SYSDDGISEVPSR.W	151 - 163
410.2474	818.4109	2	23.7	K.AHVAMYK.A	246 - 252
694.7971	1387.6480	2	27.9	K.NDSVGEHVYNVR.N	396 - 407
694.8036	1387.6480	2	20.2	K.NDSVGEHVYNVR.N	396 - 407
764.4314	763.4480	1	21.5	K.VLEYIK.S	444 - 449
764.4572	763.4480	1	22.6	K.VLEYIK.S	444 - 449
653.2654	1304.6626	2	22.5	K.AAHPEWSPAAIR.S	544 - 555
653.3392	1304.6626	2	25.5	K.AAHPEWSPAAIR.S	544 - 555
653.3468	1304.6626	2	34.1	K.AAHPEWSPAAIR.S	544 - 555
653.3529	1304.6626	2	25.6	K.AAHPEWSPAAIR.S	544 - 555
653.3554	1304.6626	2	22.4	K.AAHPEWSPAAIR.S	544 - 555
642.3013	1923.9030	3	42.6	R.SAMMTTSTALDNTQSPIR.D	556 - 573
642.3049	1923.9030	3	37.8	R.SAMMTTSTALDNTQSPIR.D	556 - 573
642.3074	1923.9030	3	34.4	R.SAMMTTSTALDNTQSPIR.D	556 - 573
642.3187	1923.9030	3	45.0	R.SAMMTTSTALDNTQSPIR.D	556 - 573
647.5990	1939.8979	3	36.8	R.SAMMTTSTALDNTQSPIR.D	556 - 573
647.6141	1939.8979	3	23.0	R.SAMMTTSTALDNTQSPIR.D	556 - 573
647.6293	1939.8979	3	35.5	R.SAMMTTSTALDNTQSPIR.D	556 - 573
647.6343	1939.8979	3	40.7	R.SAMMTTSTALDNTQSPIR.D	556 - 573
652.9795	1955.8928	3	28.4	R.SAMMTTSTALDNTQSPIR.D	556 - 573
962.9064	1923.9030	2	66.0	R.SAMMTTSTALDNTQSPIR.D	556 - 573
962.9436	1923.9030	2	77.6	R.SAMMTTSTALDNTQSPIR.D	556 - 573
962.9484	1923.9030	2	68.9	R.SAMMTTSTALDNTQSPIR.D	556 - 573
962.9574	1923.9030	2	58.8	R.SAMMTTSTALDNTQSPIR.D	556 - 573
970.8830	1939.8979	2	61.0	R.SAMMTTSTALDNTQSPIR.D	556 - 573
970.8945	1939.8979	2	58.3	R.SAMMTTSTALDNTQSPIR.D	556 - 573
970.9023	1939.8979	2	73.2	R.SAMMTTSTALDNTQSPIR.D	556 - 573
970.9029	1939.8979	2	59.8	R.SAMMTTSTALDNTQSPIR.D	556 - 573
583.6172	1747.8676	3	31.3	K.NAAATPLAMGAGHIDPNK.A	579 - 596
583.6183	1747.8676	3	21.4	K.NAAATPLAMGAGHIDPNK.A	579 - 596
583.6207	1747.8676	3	20.5	K.NAAATPLAMGAGHIDPNK.A	579 - 596
588.9408	1763.8625	3	21.3	K.NAAATPLAMGAGHIDPNK.A	579 - 596
588.9465	1763.8625	3	22.8	K.NAAATPLAMGAGHIDPNK.A	579 - 596
874.9005	1747.8676	2	75.0	K.NAAATPLAMGAGHIDPNK.A	579 - 596
874.9087	1747.8676	2	32.0	K.NAAATPLAMGAGHIDPNK.A	579 - 596
874.9139	1747.8676	2	67.4	K.NAAATPLAMGAGHIDPNK.A	579 - 596

m/z meas.	Mass calc.	z	Score	Sequence	Range
874.9140	1747.8676	2	65.3	K.NAAATPLAMGAGHIDPNK.A	579 - 596
874.9222	1747.8676	2	83.6	K.NAAATPLAMGAGHIDPNK.A	579 - 596
874.9239	1747.8676	2	82.4	K.NAAATPLAMGAGHIDPNK.A	579 - 596
882.8923	1763.8625	2	50.4	K.NAAATPLAMGAGHIDPNK.A	579 - 596
882.9093	1763.8625	2	54.5	K.NAAATPLAMGAGHIDPNK.A	579 - 596
882.9197	1763.8625	2	47.0	K.NAAATPLAMGAGHIDPNK.A	579 - 596
882.9214	1763.8625	2	55.2	K.NAAATPLAMGAGHIDPNK.A	579 - 596
664.3544	663.3592	1	25.0	R.IQEFK.R	664 - 668
664.3660	663.3592	1	27.6	R.IQEFK.R	664 - 668
410.7327	819.4603	2	26.0	R.IQEFKR.T	664 - 669
759.0184	2274.0838	3	26.3	R.TVTNLQDGTSIYTANFTPMGK.F	670 - 690
759.0236	2274.0838	3	33.2	R.TVTNLQDGTSIYTANFTPMGK.F	670 - 690
759.0296	2274.0838	3	36.8	R.TVTNLQDGTSIYTANFTPMGK.F	670 - 690
759.0353	2274.0838	3	28.5	R.TVTNLQDGTSIYTANFTPMGK.F	670 - 690
1130.0257	2258.0889	2	67.0	R.TVTNLQDGTSIYTANFTPMGK.F	670 - 690
1138.0231	2274.0838	2	48.4	R.TVTNLQDGTSIYTANFTPMGK.F	670 - 690
1138.0407	2274.0838	2	47.1	R.TVTNLQDGTSIYTANFTPMGK.F	670 - 690
516.8294	1031.6015	2	46.8	K.FKVSVVPEK.L	691 - 699
516.8328	1031.6015	2	41.8	K.FKVSVVPEK.L	691 - 699
516.8452	1031.6015	2	40.5	K.FKVSVVPEK.L	691 - 699
516.8592	1031.6015	2	29.4	K.FKVSVVPEK.L	691 - 699
757.4401	756.4382	1	27.7	K.VSVVPEK.L	693 - 699
757.4470	756.4382	1	23.6	K.VSVVPEK.L	693 - 699
757.4511	756.4382	1	20.8	K.VSVVPEK.L	693 - 699
458.3086	914.5437	2	46.5	R.SPIVATSIK.V	743 - 751
458.3099	914.5437	2	44.6	R.SPIVATSIK.V	743 - 751
458.3115	914.5437	2	46.2	R.SPIVATSIK.V	743 - 751
458.3123	914.5437	2	44.0	R.SPIVATSIK.V	743 - 751
915.5331	914.5437	1	26.0	R.SPIVATSIK.V	743 - 751
915.7249	914.5437	1	26.9	R.SPIVATSIK.V	743 - 751
426.7219	851.4137	2	25.0	K.VDPLTGHN.-	752 - 759
426.7258	851.4137	2	31.2	K.VDPLTGHN.-	752 - 759
426.7261	851.4137	2	22.6	K.VDPLTGHN.-	752 - 759

Table S5. Cleavage sites of NbSBT1 and NbSBT2 in 2F5, PG9 and human α_1-antitrypsin (A1AT). mAb CDR H3 loops (green), their tips (orange) and V_H-C_H1 linker segments (yellow) are highlighted by coloured backgrounds. The reactive center loop of A1AT is marked in cyan. Cleavage sites of NbSBT1 and NbSBT2 are indicated by red and black arrows, respectively.

2F5 (heavy chain):
RITLKESGPPLVKPTQTLTLTCSFSGFSLSDFGVGVGWIRQPPGKALEWLAIIYSDDDKRYSPSLNTRLTIT
KDTSKNQVVLVMTRVSPVDTATYFCAHRRGPTTLF↓GVPIARGPVNAMDVWGQGITVTISSTSTKGPSV
FPLAPSSKSTSGGTAALGCLVKDYFPEPVTVSWNSGALTSGVHTFPAVLQSSGLYSLSSVVTVPSSSLGT
QTYICNVNHKPSNTKVDKKVEPKSCDKTHTCPPCPAPELLGGPSVFLFPPKPKDTLMISRTPEVTCVVVD
VSHEDPEVKFNWYVDGVEVHNAKTKPREEQYNSTYRVVSVLTVLHQDWLNGKEYKCKVSNKAFPAPI
EKTISKAKGQPREPQVYTLPPSRDELTKNQVSLTCLVKGFYPSDIAVEWESNGQPENNYKTTPPVLDSD
GSFFLYSKLTVDKSRWQQGNVFSCSVMHEALHNHYTQKSLSLSPGK

2F5 (light chain):
ALQLTQSPSSLSASVGDRITITCRASQGVTSALAWYRQKPGSPPQLLIYDASSLESGVPSRFSGSGSGTEF
TLTISTLRPEDFATYYCQQLHFYPHTFGGGTRVDVRRTVAAPSVFIFPPSDEQLKSGTASVVCLLNNFYP
REAKVQWKVDNALQSGNSQESVTEQDSKDSTYSLSSTLTLSKADYEKHKVYACEVTHQGLSSPVTKSF
NRGEC

PG9 (heavy chain):
QRLVESGGGVVQPGSSLRLSCAASGFDFSRQGMHWVRQAPGQGLEWVAFIKYDGSEKYHADSVWGR
LSISRDNSKDTLYLQMNSLRVEDTATYFCVREAGGPDYRNGYNYY↓D↓FYD↓GYYNYH↓YM↓D↓VWG
KGTTVT↓VS↓SASTKGPSVFPLAPSSKSTSGGTAALGCLVKD↓YFPEPVTVSWNSGALTSGVHTFPAVLQ
SSGLYSLSSVVTVPSSSLGTQTYICNVNHKPSNTKVD↓KRVEPKSCDKTHTCPPCPAPELLGGPSVFLFPP
KPKDTLMISRTPEVTCVVVDVSHEDPEVKFNWYVDGVEVHNAKTKPREEQYNSTYRVVSVLTVLHQD
WLNGKEYKCKVSNKALPAPIEKTISKAKGQPREPQVYTLPPSREEMTKNQVSLTCLVKGFYPSDIAVEW
ESNGQPENNYKTTPPVLDSDGSFFLYSKLTVDKSRWQQGNVFSCSVMHEALHNHYTQKSLSLSPGK

PG9 (light chain):
QSALTQPASVSGSPGQSITISCNGTSNDVGGYESVSWYQQHPGKAPKVVIYDVSKRPSGVSNRFSGSKS
GNTASLTISGLQAEDEGDYYCKSLTSTRRRVFGTGTKLTVLGQPKAAPSVTLFPPSSEELQANKATLVCL
ISDFYPGAVTVAWKADSSPVKAGVETTTPSKQSNNKYAASSYLSLTPEQWKSHKSYSCQVTHEGSTVE
KTVAPTECS

A1AT:
EDPQGDAAQKTDTSHHDQDHPTFNKITPNLAEFAFSLYRQLAHQSNSTNIFFSPVSIATAFAMLSLGTKA
DTHDEILEGLNFNLTEIPEAQIHEGFQELLRTLNQPDSQLQLTTGNGLFLSEGLKLVDKFLEDVKKLYHS
EAFTVNFGDTEEAKKQINDYVEKGTQGKIVDLVKELDRDTVFALVNYIFFKGKWERPFEVKDTEEEDF
HVDQVTTVKVPMMKRLGMFNIQHCKKLSSWVLLMKYLGNATAIFFLPDEGKLQHLENELTHDIITKFL
ENEDRRSASLHLPKLSITGTYDLKSVLGQLGITKVFSNGADLSGVTEEAPLKLSKAVHKAVLTIDEKGTE
AAGAMFLE↓AIPM↓SIPPEVKFNKPFVFLMIEQNTKSPLFMGKVVNPTQK

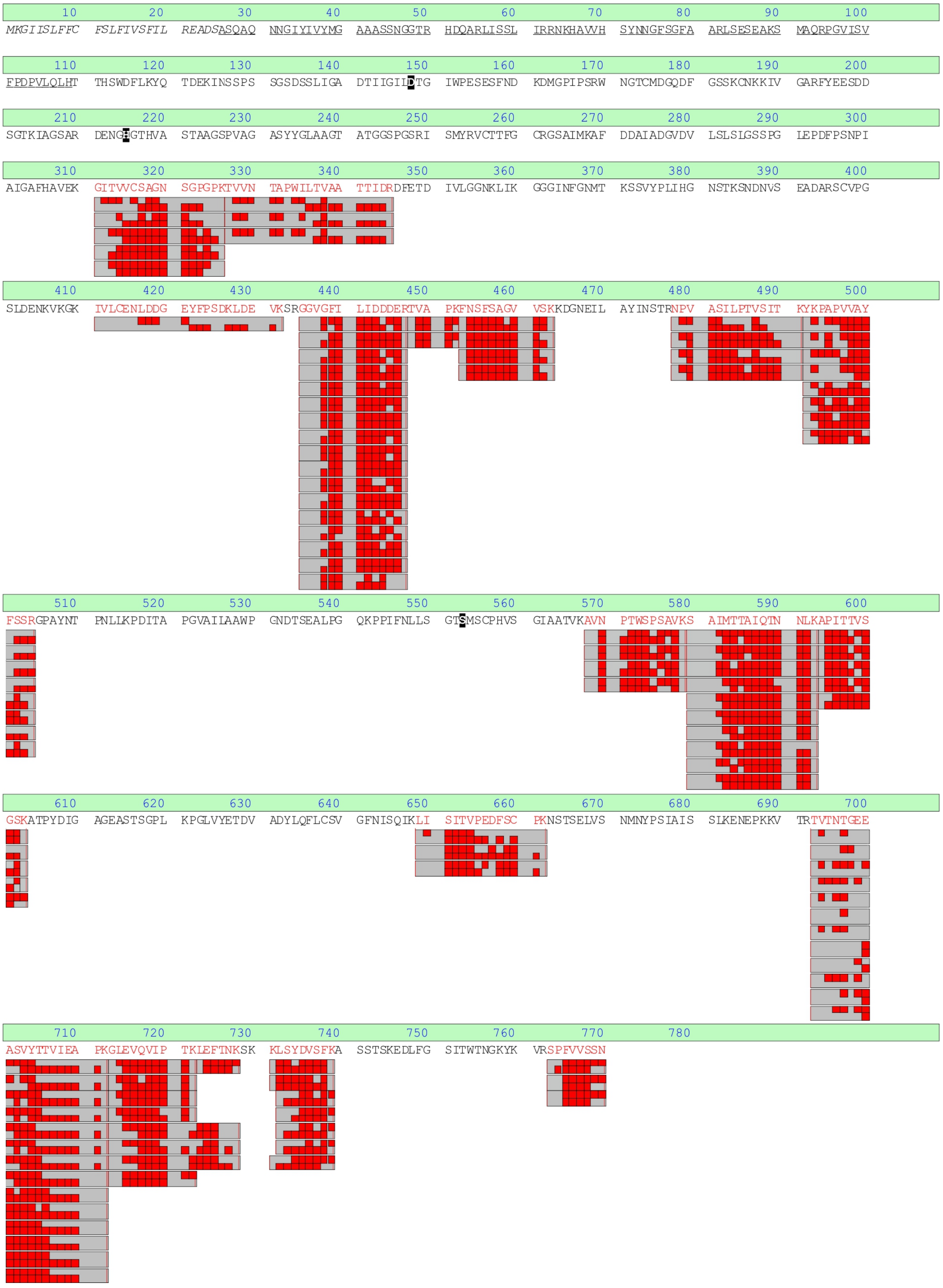

Fig. S1. Location of the identified peptides in the NbSBT1 sequence. The signal peptide is displayed in italics. The prodomain is underlined. The residues of the catalytic triad are presented as white letters on black background.

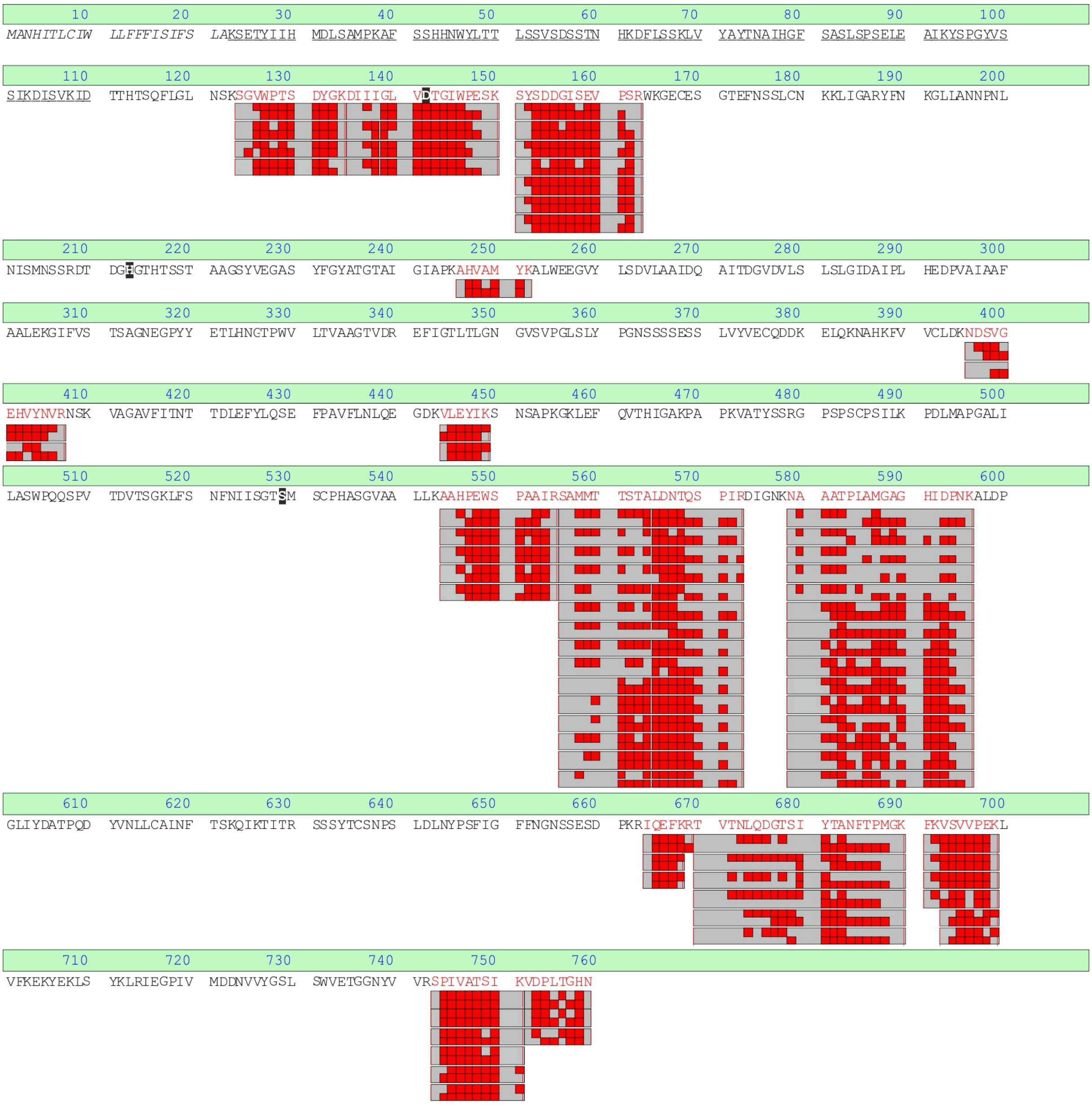

Fig. S2. Location of the identified peptides in the NbSBT2 sequence. The signal peptide is displayed in italics. The prodomain is underlined. The residues of the catalytic triad are presented as white letters on black background.

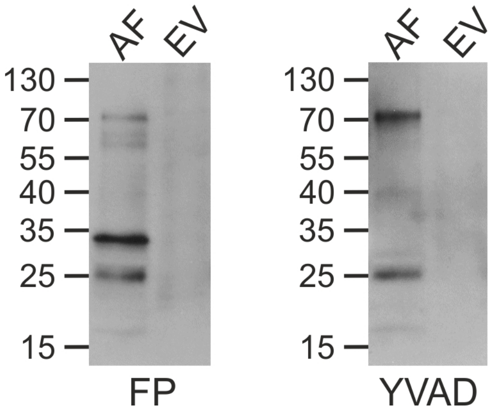

Fig. S3. Absence of subtilisin-like serine proteases in empty-vector controls. Apoplastic fluid (AF; 20 µL) and affinity-purified empty-vector control (EV; corresponding to 720 µL apoplastic fluid) were subjected to activity-based probe profiling with 100 µM FP-biotin (FP) or 10 µM biotinyl-YVAD-CMK (YVAD) for 1 h at 37°C prior to analysis by SDS-PAGE under reducing conditions and western blotting using streptavidin-peroxidase for detection. The migration positions of selected molecular mass standards are indicated, with their respective masses expressed in kDa.

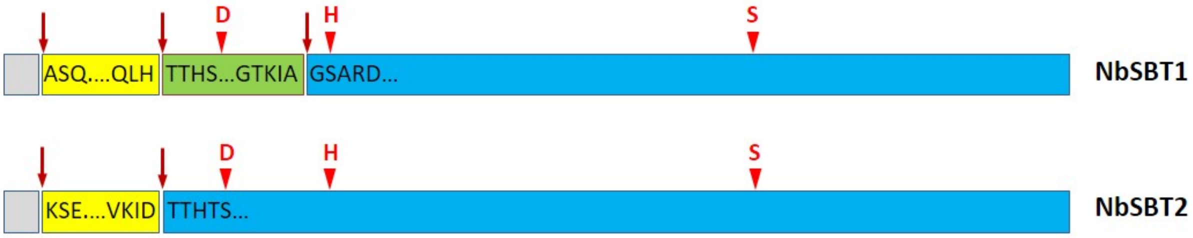

Fig. S4. Proteolytic processing of NbSBT1 and NbSBT2. Signal peptides and prodomains are displayed in grey and yellow, respectively. Mature NbSBT1 is processed into non-covalently associated light (green) and heavy (blue) chains. Cleavage sites are labelled with red arrows. The positions of the active-site residues are indicated with red arrowheads.

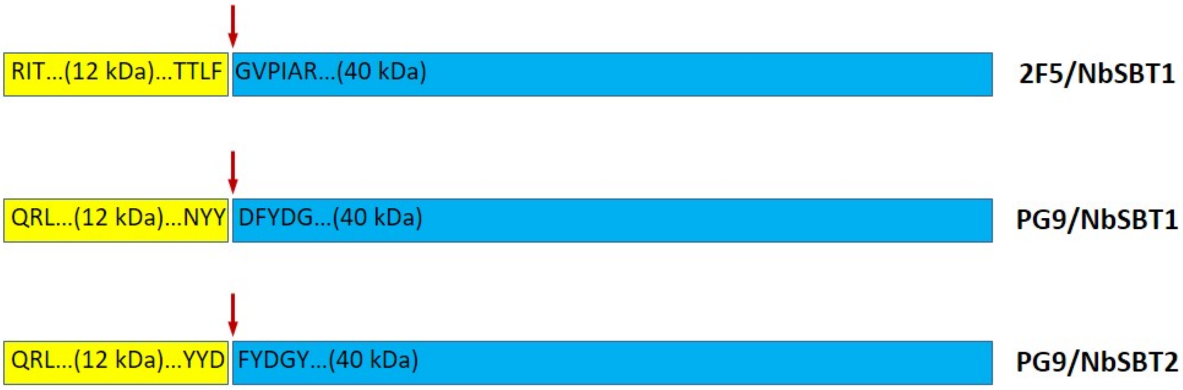

Fig. S5. Processing of 2F5 and PG9 by NbSBT1 and NbSBT2. Only the most N-terminal cleavage events are shown for PG9.
